# Structures and Dynamics of Dengue Virus Nonstructural Membrane Proteins

**DOI:** 10.3390/membranes12020231

**Published:** 2022-02-17

**Authors:** Qingxin Li, Congbao Kang

**Affiliations:** 1 Guangdong Provincial Engineering Laboratory of Biomass High Value Utilization, Institute of Biological and Medical Engineering, Guangdong Academy of Sciences, Guangzhou 510316, China; 2 Experimental Drug Development Centre, Agency for Science, Technology and Research, 10 Biopolis Road, #5-01, Singapore 138670, Singapore

**Keywords:** dengue virus, membrane protein, membrane topology, drug discovery, nonstructural proteins, antiviral development, protein dynamics, NMR spectroscopy

## Abstract

Dengue virus is an important human pathogen threating people, especially in tropical and sub-tropical regions. The viral genome has one open reading frame and encodes one polyprotein which can be processed into structural and nonstructural (NS) proteins. Four of the seven nonstructural proteins, NS2A, NS2B, NS4A and NS4B, are membrane proteins. Unlike NS3 or NS5, these proteins do not harbor any enzymatic activities, but they play important roles in viral replication through interactions with viral or host proteins to regulate important pathways and enzymatic activities. The location of these proteins on the cell membrane and the functional roles in viral replication make them important targets for antiviral development. Indeed, NS4B inhibitors exhibit antiviral activities in different assays. Structural studies of these proteins are hindered due to challenges in crystallization and the dynamic nature of these proteins. In this review, the function and membrane topologies of dengue nonstructural membrane proteins are presented. The roles of solution NMR spectroscopy in elucidating the structure and dynamics of these proteins are introduced. The success in the development of NS4B inhibitors proves that this class of proteins is an attractive target for antiviral development.

## 1. Introduction

Dengue virus (DENV) and other viruses such as Zika virus and West Nile virus are members of the *Flaviviridae* family. DENV is an important human pathogen and contains four serotypes (DENV-1–4) which can be transmitted by mosquitos. DENV threatens populations worldwide and causes over 390 million human infections annually [1]. Although dengue infection can result in mild symptoms such as fever and joint pain, the infection can also induce dengue fever, dengue hemorrhagic fever and dengue shock syndrome [1]. Therefore, it is important to develop therapies against viral infection. Vaccines have been developed, although application of vaccines is limited due to the possible risk [2,3]. Efforts have been made to develop chemical therapies by targeting viral proteins [2,4,5,6,7,8,9,10,11,12,13,14,15,16,17,18]. No approved drugs are available for viral treatment due to challenges in drug discovery [19].

**Figure 1:**
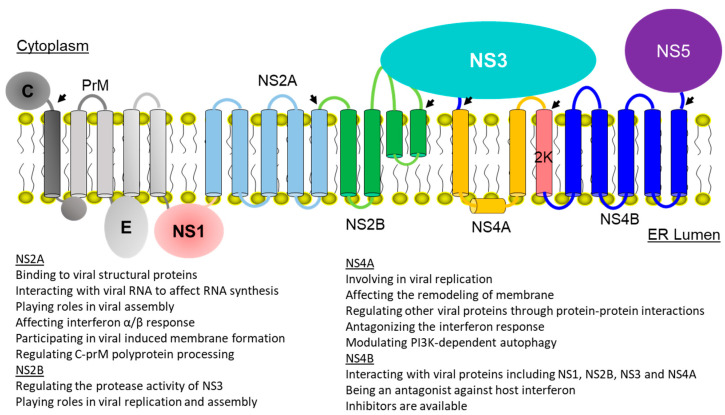
Organization and membrane topologies of dengue proteins. The viral proteins are highlighted in different color. The NS2B-SN3 protease cleavage sites are indicated as arrows. The functions of NS2A, NS2B, NS4A and NS4B are listed.

The viral genome is a positive-sense, single stranded RNA with a size of 11 kb that contains one open reading frame. The viral genome encodes one polyprotein that can be processed into three structural proteins—capsid protein (C), membrane protein (M) and envelope protein (E)—and seven nonstructural proteins—NS1, NS2A, NS2B, NS3, NS4A, NS4B and NS5 (Figure 1) [20]. The structural proteins are assembled into virions with the viral genome, and the nonstructural proteins play important roles in viral RNA replication, virion assembly and evasion of host immune response through their enzymatic activities or protein–protein interactions [5,21,22,23,24].

The functions of the seven nonstructural proteins have been well characterized. Three nonstructural proteins (NS1, NS3 and NS5) are water soluble and four proteins (NS2A, NS2B, NS4A and NS4B) are membrane bound [25,26]. NS3 harbors protease and helicase activities in its N-terminal and C-terminal domains [27,28,29,30,31,32]. NS5 possesses RNA-dependent RNA polymerase (RdRp) activity and RNA methyltransferase (MTase) activity [33]. Biochemical assays to evaluate the activity of NS3 and NS5 have been developed, which makes it possible to develop potent inhibitors [16,34,35,36,37]. Despite progress made in developing inhibitors of NS3 and NS5 [9,33,38,39,40], there is no compound reaching clinical studies [41,42,43]. One of the challenges is the hydrophilic nature of the active sites, which hinders the development of hydrophobic small-molecule inhibitors [39,40]. Some membrane bound nonstructural proteins are considered in drug discovery and quite a few potent inhibitors of NS4B have been developed [44,45,46]. In this review, the functions and membrane topologies of dengue nonstructural membrane proteins are described. The progress in drug discovery against some membrane proteins is introduced. Understanding the folding and dynamics of these proteins will be useful for developing potent viral inhibitors.

## 2. Membrane Topologies and Functions of Viral Membrane Proteins

One of the characteristics of these viral membrane proteins is that all proteins contain various amounts of transmembrane helices which are indispensable for their location on the cell membrane [47,48,49,50,51]. Therefore, the folding of these proteins in vitro requires the presence of membrane mimicking systems such as micelles, bicelles and lipid bilayers [52,53,54,55,56]. Dengue membrane proteins do not possess any enzymatic activities, which makes it challenging to explore their folding and function in vitro. Despite such a challenge, the membrane topologies of these membrane proteins have been determined using different strategies. In addition, it is possible to explore the structures and dynamics of these proteins in vitro using structural tools such as solution NMR spectroscopy because of their molecular weights.

### 2.1. Dengue NS2A

**Figure 2:**
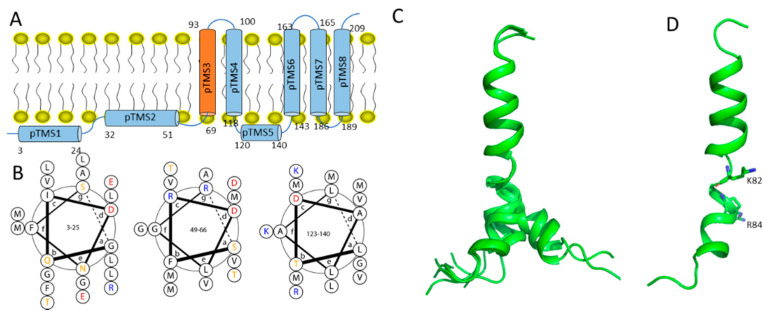
Membrane topology of NS2A and residues critical for the function of NS2A. (**A**) Membrane topology of NS2A. The transmembrane helix with structure determined using NMR is highlighted in brown. (**B**) Helix view of the three non-transmembrane helices. Helical wheels of these sequence are plotted using DrawCoil 1.0 (https://grigoryanlab.org/drawcoil/ (accessed on 14 February 2022)). (**C**) NMR structures of pTMD3 in organic system. (**D**) One structure of pTMS3. The structure (PDB id 2M0S) is shown. The structures were made using PyMOL (https://pymol.org/2/ (accessed on 14 February 2022)). The two positively charged residues in the helix are shown in sticks. More details can be obtained from the reference [57].

Dengue NS2A contains more than 200 residues with a molecular weight of approximately 22 kDa (Figure 2A). NS2A was shown to have five transmembrane helices with N-terminal 68 residues localizing in the endoplasmic reticulum (ER) lumen and the C-terminus consisting of residues 210–218 localizing in the cytosol [57]. NS2A plays important roles in viral replication and pathogenesis through its interactions with RNA and proteins. It is an important component of the replication complex on the cell membrane. Studies have shown that NS2A interacts with RNA through binding to 3′ UTR, which is critical for RNA synthesis [57,58]. Point mutation in NS2A affects both RNA synthesis and virus production, confirming its roles in viral RNA synthesis and virion assembly [57]. In addition to interacting with NS3, yellow fever virus NS2A was shown to be important for virion assembly via a basic cluster localized at the N-terminus [25]. Mutations in some residues in NS2A resulted in viruses with efficient RNA replication while no infectious viruses were produced. Such results suggest its roles in virion assembly [25]. NS2A plays important roles in virus assembly through its involvement in the biogenesis of the virus induced membranes [26]. In addition, NS2A alone is able to suppress the interferon α/β response, which affects viral replication and induces apoptosis in infective cells [59,60].

Dengue NS2A contains five transmembrane helices based on several biochemical assays (Figure 2A). Although no tertiary structure of the entire NS2A is available, the membrane topology of NS2A provides useful insights into its function. The five transmembrane segments of NS2A cause the N- and C-termini to be located at the two sides of the ER membrane. Releasing N- and C-terminal ends requires different proteases, including host signalase and viral NS2B-NS3 protease. The availability of several helices at the ER lumen site makes them able to interact with different proteins. Three amphipathic helices are present in NS2A (Figure 2B). These helices might interact with cell membranes and other molecules due to the presence of a hydrophobic and a hydrophilic interface. The first amphipathic helix plays a role in protein–protein interactions. A recent study showed that G11A mutation in dengue-2 NS2A abolished virion assembly without affecting RNA synthesis. The distributions of E, C and prM proteins were affected in the G11A transfected cells. C protein was present in both nucleus and cytoplasm in viral transfected cells while it was present mainly in cytoplasmic in G11A mutant cells. PrM protein was not observed in the ER region in G11A infected cells. Although the transmembrane domains of PrM were demonstrated to be critical for binding to NS2A, coimmunoprecipitation experiments showed that G11A NS2A pulled down more prM (~2.5 folds) than wile type NS2A did [61]. Positively charged residues between the first and second transmembrane helices are critical for RNA binding and virion assembly. Viral mutants at several positions such as R94, R95 and K99 exhibited lower RNA binding affinities, which is lethal for virion production [61].

The solution structure of the first transmembrane region of dengue NS2A was determined using solution NMR spectroscopy (Figure 2C,D) [57]. Although the structure was determined in a system containing organic solvents, the result provides useful information to understand the function NS2A, which reveals that there are two helical segments in the transmembrane region separated by a proline residue at position 85. Interestingly, the mutation of P85 does not affect viral RNA synthesis while R84 is critical for viral replication. The residues in transmembrane domains are usually hydrophobic. The charged residue in a transmembrane domain can play a role in transmembrane helix packing [62], which might occur within NS2A transmembrane helices or in other viral membrane proteins. Although no structural studies have been carried out to understand the roles of R84 in helix packing, R84E mutation was shown to affect RNA synthesis [57].

### 2.2. Dengue NS2B

**Figure 3:**
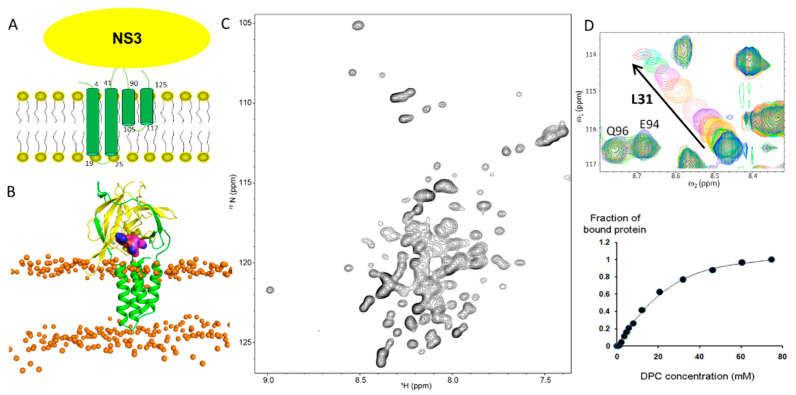
Structural studies on dengue NS2B. (**A**) Membrane topology of NS2B on the cell membrane. (**B**) Model of NS2BFL-NS3 pro. The native form of protease containing full-length NS2B and NS3 protease domain (NS3pro) is shown. NS2B and NS3 are highlighted in green and yellow, respectively. The P1–P4 residues are shown as a sphere structure. (**C**) The ^1^H-^15^N-HSQC spectrum of full-length NS2B in detergent micelles. This figure is obtained from [48] with permission. (**D**) Binding of protease domain to micelles revealed by NMR spectroscopy. Residues such as L31 from NS3 are critical for binding to the membrane. This figure was obtained from [86] with permission.

Dengue NS2B is a small membrane protein with a molecular weight of approximately 15 kDa (Figure 3A). The well-known function of NS2B is to regulate the activity of NS3 protease. NS2B acts as a cofactor of NS3 protease domain by forming a tight complex via one of its hydrophilic regions consisting of approximately 40 amino acids (Figure 3B). NS2B contains four transmembrane helices which do not bind to NS3 while these helices are required for NS3 to approach the membrane where the C-terminal region of NS3 can play important roles in viral replication [63,64].

The cofactor roles of NS2B on NS3 can be summarized into the following sections. First, the cofactor region is formed by approximately 40 residues between the second and third transmembrane helices of NS2B and is critical for the folding and the protease activity of the NS3 protease domain. The protease domain alone was not well folded when it was expressed in bacterial cells. To determine the structure of NS2B-NS3, artificial constructs containing the NS2B cofactor region and NS3 protease domain were utilized [34,65,66,67]. The NS2B cofactor region and NS3 protease domain form a stable complex which can be purified for biochemical and structural studies. Second, the cofactor region of NS2B forms the partial active site of the protease. The NS3 protease region contains a catalytic triad comprising H51, D74 and S135. The C-terminal part of the NS2B cofactor region (residues 75–87) forms a β hairpin structure interacting with the substrate. The protease lacking this hairpin region exhibits almost no enzymatic activity [68]. Based on X-ray and NMR structural studies, it is obvious that the N-terminal portion of the NS2B cofactor region binds tightly to the N-terminal region of NS3 [34,65,67,69,70,71,72]. The C-terminal portion can form a β hairpin structure participating in substrate binding, which results in an active protease-closed conformation. Studies also show that the C-terminal portion of the cofactor region can stay away from the active site and is dynamic in solution, which results in an inactive protease-open conformation [73,74,75]. Although studies have shown that the closed conformation should be utilized in structure-based drug design, conformational exchanges are present at the C-terminal portion of the NS2B cofactor region [76,77,78]. Lastly, the cofactor role of NS2B on NS3 can also be attributed to bringing NS3 close to the cell membrane where NS3 can be part of the replication complex and play other important roles such as helicase activity and forming a complex with NS4B. The NS2B might also play a role in forming oligomers. It was revealed by one study that NS2B-NS3 could form oligomers based on gel filtration profiling and cross-linking experiments while the function of such a complex needs to be explored [79].

In addition to being a cofactor of NS3 protease, NS2B plays important roles in virus replication and assembly by participating in molecular interactions with different molecules [80]. NS2B is present in the replication complex on the ER membrane and involves protein–protein interactions with virus non-structural or host proteins. NS2B may bind to dsRNA, implying its roles in the replication complex [81]. A study has shown that the NS2B of Japanese encephalitis virus (JEV) is important for viral replication. Mutation of residues in the transmembrane domain attenuated or destroyed the viral RNA synthesis [82]. In addition, the hydrophobic regions of NS2B may be responsible for interacting with the host membrane or involved in protein–protein interactions, which contributes to its ability to alter membrane permeability. Overexpression of NS2B in bacterial cells results in changes of permeability [83]. A recent study shows that NS2B alone is able to degrade cyclic GMP-AMP synthase (cGAS) in the absence of NS3 [84]. As cGAS is functional as a cytosolic DNA sensor to activate the ER host protein STING for type I interferon (IFN) signaling [85], NS2B is vital in reducing the host’s innate response upon viral infection to promote viral replication and disease.

The structure of the NS2B cofactor region in complex with NS3 has been thoroughly studied [34,65,66]. Its N-terminal region forms a β-strand structure interacting with a strand from NS3 and its C-terminal part forms a β-hairpin or is unstructured in the presence or absence of a substrate or an inhibitor. Based on the conformational changes of the NS2B cofactor region, NS2B-NS3 is considered to form open and closed conformations [68]. Although the changes can be suppressed by using different artificial constructs or adding a substrate, efforts have been made to develop inhibitors stabilizing the open form to suppress viral protease activity [70,87]. The NS2B cofactor region is unstructured and flexible in the absence of NS3, which has been confirmed by solution NMR study [71]. Similar conformation was observed in the full-length NS2B revealed by NMR spectroscopy [48]. In the study, full-length dengue NS2B was reconstituted in detergent micelles (Figure 3C). Based on the residue specific resonance assignments, the secondary structures of residues in NS2B were obtained. NS2B is comprised of four transmembrane helices and the cofactor region is unstructured even in detergent micelles. The four helices may form a helix bundle in solution according to analyses of the chemical shifts of backbone resonances while further studies are required to confirm this prediction [48]. The model of the native form of protease containing full-length NS2B and NS3 protease domain (NS3pro) has been predicted [88]. NS3pro has a region binding to the cell membrane, which has been confirmed by NMR spectroscopy using a detergent system (Figure 3D) [86,89]. Although a construct harboring full-length NS2B and NS3 protease domain can be purified from bacterial cells [52,53,90], no structure of such a complex was obtained so far, which may be due to the challenges in crystallization.

### 2.3. Dengue NS4A

**Figure 4:**
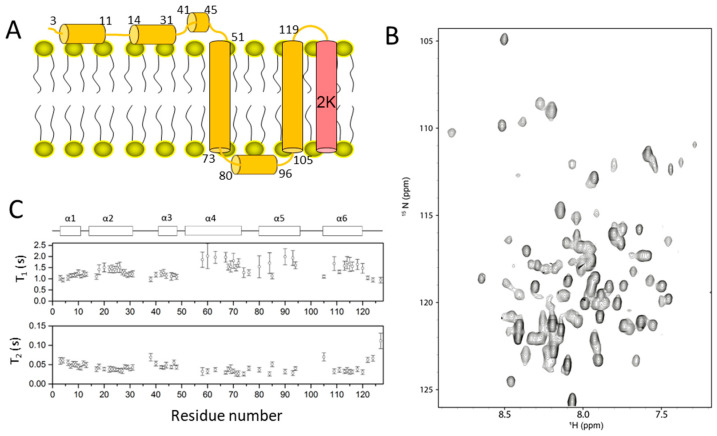
Structural studies on dengue NS4A. (**A**) Membrane topology of NS4A. The helices identified by NMR spectroscopy are shown as cylinders. (**B**) The ^1^H-^15^N-HSQC spectrum of full-length NS4A in micelles. (**C**) Dynamic analysis of NS4A. The dynamics of NS4B shows that these helices are amphipathic or transmembrane helices due to their different relaxation rates. This figure is obtained from the reference [50] with permission.

NS4A is a membrane protein with a molecular weight of approximately 16 kDa (Figure 4A). There is a 2K sequence at the C-terminus of NS4A. The 2K sequence is separated from NS4A through the protease cleavage and it serves as a signal peptide for the translocation of NS4B to ER lumen [91,92]. The N-terminal region of NS4A may interact with the cell membrane via some hydrophobic residues [93,94]. This region might play a role in regulating the curvature of the cell membrane [94,95]. The residues at the N-terminal region are critical for protein oligomerization, and mutations in this region can stop viral replication [96,97]. The C-terminal region of NS4A consists of three transmembrane helices forming a U-shaped structure, which makes the N- and C-termini of NS4A face the cytoplasm.

Several studies have been performed to explore the function of NS4A, and it is suggested that both mature NS4A and a part of the viral polyprotein cleavage product NS4A-2K-NS4B play important roles in viral replication. The function of NS4A has been summarized recently [98]. First, NS4A localizes on the ER membrane, serving as an important component of the viral replication complex consisting of viral proteins, dsRNA and host proteins. Based on studies using West Nile virus NS4A, the N-terminal region of NS4A is shown to play important roles in the assembly of a viral replication complex to the cholesterol-rich region on the ER membrane [99]. The C-terminal part of NS4A is critical for the formation of the viral replication complex as mutations in the PEPE motif affect the complex formation, resulting in suppression of viral replication [100,101]. Dengue NS4A has been shown to interact with vimentin through its residues in the N-terminal region, which is critical for precisely anchoring the viral replication complex to the perinuclear membrane [102]. Second, NS4A plays a vital role in membrane remodeling, which is a key step in cells infected by flaviviruses. Proliferation of ER membranes and formation of double-membrane vesicles are critical steps for viral replication [103,104,105,106]. The N-terminal region of NS4A is indispensable for changing the structure of the membrane through direct interactions. Several mechanisms have been proposed to illustrate the roles of NS4A in altering membranes. The presence of an amphipathic helix is necessary for binding to the membrane, which plays a role in inducing membrane curvature [94,95,107]. Homo-oligomerization of NS4A may be important for its roles and the amphipathic helices in the N-terminal region are critical for such functions because point mutations in these helices reduce protein oligomerization [96,108,109]. It has been noted that residues of the N-terminal region of NS4A are indispensable for NS4A stability. Point mutations of some conserved residues within this region affect membrane proliferation [101,110]. Such observed effects might be due to alterations in membrane binding after mutation. Third, NS4A participates in interactions with other viral proteins to regulate viral replication [97]. A viral polyprotein cleavage fragment NS3-NS4A was detected during viral polyprotein processing [111,112]. A study using a helicase domain of NS3 fused with the N-terminal region of NS4A demonstrated that NS4A does not affect the oligonucleotide duplex unwinding rate, but NS4A can affect the ATPase activity. This result suggests that NS4A might serve as a cofactor of the NS3 helicase to affect the ATPase activity [113]. Further studies showed that direct physical interactions between NS1 and NS4A-2K-NS4B cleavage intermediate were critical for viral RNA replication [114,115]. NS4A and NS4B interactions are also indispensable for the function of both proteins [116,117]. Lastly, NS4A plays important roles in viral replication and pathogenesis by interacting with host proteins or affecting important pathways. The roles of NS4A in interferon response and autophagy have been described in a recent review [98]. NS4A has been shown to play a role in antagonizing the IFN response in humans [118,119]. NS4A is able to modulate PI3K-dependent autophagy critical for viral replication [120]. NS4A binds directly to reticulon (RTN) protein RTN3.1 A, which is necessary for the stability of NS4A and membrane remodeling [121].

The structures of the N-terminal domain of NS4A and full-length NS4A have been investigated [50,95,122]. Although the three-dimensional structure of NS4A was not obtained, the available secondary structure of this protein provides useful information to understand its function. Two helices were observed in the N-terminal domain when this domain alone was subjected to NMR studies [95]. A short helix containing residues 41–48 was identified when the full-length NS4A was used in an NMR analysis [109]. Based on accumulated studies, NS4A is comprised of six helices. The three helices from the N-terminal region might bind to the cell membrane while the three helices from the C-terminal region form a U-shaped structure in the cell membrane (Figure 4A). All these helices form rigid structures in solution, which was confirmed by the dynamic studies (Figure 4B,C). The available NMR spectra of NS4A made it possible to probe protein–protein interactions in vitro.

### 2.4. Dengue Virus NS4B

**Figure 5:**
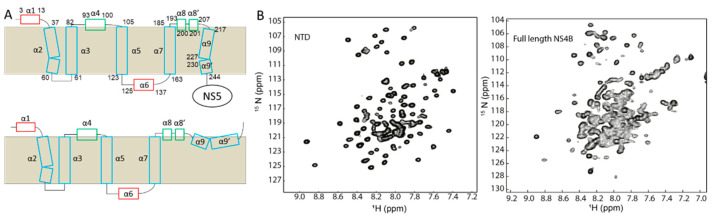
Structural studies on dengue NS4B. (**A**) Membrane topology of NS4B based on the NMR studies. The secondary structures of NS4B were determined. (**B**) ^1^H-^15^N-HSQC spectra of the N-terminal domain of NS4B and the full-length NS4B in detergent micelles. This figure is obtained from the reference [47,49] with permission.

Dengue virus NS4B is a membrane protein consisting of 248 residues with a molecular weight of approximately 27 kDa (Figure 5A). It is an important component of the viral replication complex on the ER membrane and colocalized with NS3 and viral double-stranded RNA [123,124,125,126]. The 2K peptide preceding NS4B can be released through cleavage by a host signalase, and its C-terminus release is achieved via the NS2B-NS3 protease. NS4B does not possess any enzymatic activities and plays important roles in viral replication and affects host immunity through protein–protein interactions [7,46,127,128,129].

NS4B forms homodimers mediated through its cytosolic loop (residues 129–165) and its C-terminus [123]. NS4B has been shown to interact with other viral proteins, including NS1, NS2B, NS3 and NS4A [115,130,131,132]. The interactions are critical for viral replication. The interaction between NS4A and NS4B is requisite for viral replication [116]. The long loop of NS4B is indispensable for binding to the NS3 helicase, which can enhance the dsRNA unwinding activity [132]. Disrupting protein–protein interactions can serve as a strategy to develop novel antivirals. Indeed, a recent study identified an inhibitor that exhibited pan-dengue activity [45]. NS4B also plays important roles in affecting the host’s immune response. Dengue virus NS4B has been shown to be an antagonist against host type-I interferon, which is mediated via affecting phosphorylation of STAF1 [60,133].

The membrane topology of NS4B was obtained using biochemical analysis. In the model, NS4B is comprised of five helices [134]. The N-terminal region consists of two membrane-binding helices. The C-terminal part of NS4B contains three transmembrane helices. This model is helpful for interpreting the function of NS4B. Recombinant dengue virus NS4B was purified and reconstituted in detergent micelles [47,49]. Although no crystal structure of NS4B is obtained, it is possible to evaluate its structures using solution NMR spectroscopy (Figure 5B). Secondary structural analysis revealed that NS4B is comprised of eleven helices in solution (Figure 5A). Compared with the model built from biochemical analysis, the NMR study identified some more short helices [47,49]. In addition, the transmembrane helix at the C-terminus contains two short helices, which may be critical for the functioning of NS4B. Further dynamics study demonstrated that the five helices identified in the biochemical assay exhibited similar dynamic values, suggesting that these helices are buried in micelles [47,49]. The available NMR spectrum of NS4B is useful for understanding its structure and probing binding with NS4B inhibitors [135].

## 3. Dengue Membrane Proteins as a Drug Target

Dengue nonstructural membrane proteins are critical for viral replication, virion assembly and affecting the host’s immune response [51]. Therefore, they are promising targets for developing antivirals. As these membrane proteins do not harbor any enzymatic activities, it is challenging to identify specific inhibitors using a biochemical assay. Despite challenges encountered for such targets, there are several inhibitors available against these membrane proteins.

### 3.1. NS2A Inhibitors

There is no dengue NS2A inhibitor available to date due to its lack of enzymatic activity. A calmodulin inhibitor W7 was reported to exhibit anti-dengue activity in a cell-based assay. The mechanism of action of this inhibitor may be due to its interaction with calmodulin which interacts with NS2A’s calmodulin binding motif [136]. Although W7 could disrupt calmodulin and NS2A interactions, no compound optimization was carried out to improve its potency. In addition to identifying inhibitors disrupting protein–protein interactions, affecting NS2A and membrane interactions is of great interest in drug discovery. Several regions of NS2A were found to bind specifically to the cell membrane [137]. For example, peptide dens25 can insert into membranes and interact specifically with negatively-charged phospholipids [138]. Nordihydroguaiaretic acid (NDGA) was shown to reduce viral yield through reduction of lipid droplets and resulted in the dissociation of C protein from lipid droplets [51,139]. Disrupting protein–membrane interactions might be a strategy to develop NS2A inhibitors while further compounding optimization will be challenging due to the lack of a binding pocket in NS2A. A structural model of NS2A was proposed, and NS2A might form a helix bundle, which makes it able to develop small molecules to affect the function of NS2A [80]. As no recombinant dengue NS2A protein is available, the strategy of developing NS2A inhibitors would rely on cell-based assays. Obtaining a high-result structure of NS2A and understanding its protein–protein interaction network will be very helpful for designing its inhibitors.

### 3.2. NS2B Inhibitors

NS2B is critical for the protease activity of NS3. As the C-terminal part of NS2B harboring P1-P4 residues binds weakly to NS3 [140], the potency of the peptide was improved by adding a warhead to the peptide sequence to form a covalent bond with S135 of the NS3 [40,42,43,70,141,142,143] and changing the length of the NS2B peptide to reduce the molecular weight [40,70]. Due to the exchanges present in the NS2B-NS3 protease complex, allosteric inhibitors have been developed and exhibited potent activity against viral replication [144,145,146,147]. It has been noted that all these inhibitors are considered as NS3 inhibitors even though some of the inhibitors were derived from NS2B and bind to several residues in NS2B. There are no NS2B inhibitors available so far. One strategy to obtain NS2B binders is to use the native viral protease construct containing full-length NS2B and NS3 protease domain or the full-length NS2B protein [52,56,90]. Like other viral membrane proteins, NS2B alone does not have any enzymatic activities; biophysical methods are needed to probe protein–ligand interactions in hit identification and compound optimization. The four helices may form a helix bundle in solution based on structural prediction using chemical shifts, and obtaining the structure of full-length NS2B will be very helpful for understanding the feasibility of developing NS2B inhibitors [48]. The availability of recombinant NS2B makes it possible to identify the NS2B binders through a target-based drug discovery approach such as fragment-based drug design [48,53,148,149].

### 3.3. NS4A Inhibitors

NS4A consists of three transmembrane helices forming a U-shaped structure which lacks a pocket binding to small molecules. Therefore, developing peptidic inhibitors is feasible to affect its function. The amphipathic helices or transmembrane helices of NS4A might be utilized as a starting point for further development [150,151]. In addition, preventing maturation of NS4A is of great interest in drug discovery. A peptide sequence from the C-terminus of NS4A was proposed to serve as a template for developing inhibitors to suppress cleavage of NS4A-2K [51,100].

### 3.4. NS4B Inhibitors

Several NS4B inhibitors have been reported. These small molecules were identified using cell-based assays and exhibited activities against viral replication [7]. One study shows the identification of an inhibitor containing a spiropyrazolopyridone core and the resulting inhibitor is able to inhibit dengue 2 and dengue 3 serotypes. An escaping mutant containing a mutation of NS4B at position 63 was identified, suggesting that the inhibitors bind to NS4B directly [46,152]. A lead compound NITD-688 was developed and shown to exhibit activity against four dengue serotypes. This compound also demonstrated efficacy in animal models. Resistance mutation analysis revealed that NS4B is the target of this inhibitor and further NMR study confirmed the molecular interactions [44]. The potency of this inhibitor suggests that it can be a candidate for further development. Another potent inhibitor, JNJ-A07, was developed by optimizing hits from a large-scale cell-based anti-DENV-2 screen. Pan-genotype and pan-serotype activities were observed for this compound as it was active against all four dengue serotypes [45]. Analyzing drug-resistant strains revealed that NS4B is the main target of this compound. Further study suggested that JNJ-A07 was able to disrupt molecular interactions of NS4B with NS3. This inhibitor also demonstrated a strong in vivo potency in mice. The research into developing the NS4B inhibitor also proves that the drug repurposing approach can play an important role in drug discovery due to the similar structure of JNJ-A07 to other patented compounds [153].

## 4. Perspectives

**Figure 6:**
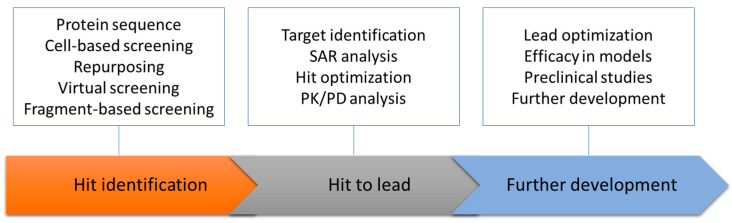
A simplified flowchart to develop antivirals by targeting these membrane proteins. The key step is to identify hits binding to these proteins, which is challenging as no biochemical assays are available. Deconvolution of hits identified from cell-based assays is needed for understanding the mechanism of action. Using biophysical methods to explore protein–ligand interactions will be useful when recombinant proteins are available.

Efforts have been made to develop inhibitors against NS2B-NS3 protease, NS3 helicase and NS5, but no inhibitors are available for clinical studies [2,8,9,10,15,29,30,37,154]. Several reviews have summarized the challenges in developing inhibitors against these viral proteins with enzymatic activities [39,155,156,157]. These small non-structural membrane proteins represent a novel class of targets for antiviral development [19,51,98,158]. Their high hydrophobicity makes it feasible for them to bind to small molecules suitable for further development or serves as a starting point for developing peptidic inhibitors, although challenges remain. The development of NS4B inhibitors encourages researchers to explore inhibitors targeting this type of target without any enzymatic activities [45,153,159]. The cell-based assays play important roles in hit identification targeting this type of target. The targets of the identified hits can be confirmed by generating resistance virus and sequence analysis. It has been noted that this approach is time consuming. Target-based drug discovery approaches can be utilized in these targets when recombinant proteins in membrane systems for in vitro studies are available [135,148,160] (Figure 6). The available recombinant NS2B, NS4A and NS4B proteins make it possible to identify hits binding specifically to these proteins, understand the structure–activity relationship of the inhibitors and evaluate compound binding affinities using biophysical methods. These proteins will serve as validated targets for antiviral development. Structural studies of these proteins will be valuable for understanding the mechanism of action of known inhibitors, performing virtual screening and optimizing the identified hits.
